# Two Clinical Cases

**Published:** 1890-05

**Authors:** Robert Farley

**Affiliations:** Phœnixville, Pa.


### TWO CLINICAL CASES.

Robert Farley, M. D., Phœnixville, Pa.

In an epidemic of pertussis during the past winter, I have experienced much difficulty in finding the homœopathic remedy. Finally I noticed that the paroxysms of cough were preceded by a fretful, anxious condition in some cases for fifteen minutes. With this symptom in the cases I was led to prescribe Cupr.-met.[30] with gratifying results, thus verifying the symptom, "anxious before cough."

In June of 1888, I was called to relieve a patient who had for many years been a sufferer from "hay fever." He suffered terribly during the summer and autumn. He would have sudden spells of suffocation at night, causing him to jump up, and only relieved by eating; would hastily eat anything he could get his hands on that was edible. In studying the case, I decided that Sang.-c. was the most similar remedy, and gave it in the thirtieth potency with almost instant relief, and there has been no return of his trouble to date, having passed through two summers. I report this because Sang. is not credited with the above relief from eating; Graph. being the only remedy having it that I could discover.

---

